# Arbuscular mycorrhizal fungi induce the expression of specific retrotransposons in roots of sunflower (*Helianthus annuus* L.)

**DOI:** 10.1371/journal.pone.0212371

**Published:** 2019-02-19

**Authors:** Alberto Vangelisti, Flavia Mascagni, Tommaso Giordani, Cristiana Sbrana, Alessandra Turrini, Andrea Cavallini, Manuela Giovannetti, Lucia Natali

**Affiliations:** 1 Department of Agriculture, Food, and Environment, University of Pisa, Pisa, Italy; 2 CNR, Institute of Agricultural Biology and Biotechnology UOS Pisa, Pisa, Italy; University of Helsinki, FINLAND

## Abstract

Retrotransposon expression during arbuscular mycorrhizal (AM) fungal colonisation of sunflower roots (*Helianthus annuus*) was analysed using Illumina RNA-Seq, in order to verify whether mycorrhizal symbiosis can activate retrotransposable elements. Illumina cDNA libraries were produced from RNAs isolated from the roots of sunflower plants at 4 and 16 days after inoculation with the AM fungus *Rhizoglomus irregulare* and from their respective control plants. Illumina reads were mapped to a library of reverse transcriptase-encoding sequences, putatively belonging to long terminal repeat retrotransposons of *Gypsy* and *Copia* superfamilies. Forty-six different reverse transcriptase sequences were transcribed, although at a low rate, in mycorrhizal or control roots and only four were significantly over-expressed at day 16, compared with control roots. Almost all expressed or over-expressed sequences belonged to low-copy elements, mostly, of the *Copia* superfamily. A meta-analysis, using publicly available Illumina cDNA libraries obtained from sunflower plants treated with different hormones and chemicals, mimicking stimuli produced by abiotic and biotic stresses, was also conducted. Such analyses indicated that the four reverse transcriptase sequences over-expressed in mycorrhizal roots were explicitly induced only by AM symbiosis, showing the specificity of AM stimuli compared to that of other fungal/plant interactions.

## Introduction

Arbuscular mycorrhizal (AM) fungi are beneficial symbionts of roots of most plant species, including many cultivated plants. AM fungi (AMF) are beneficial obligate biotrophic microorganisms, represent essential elements of soil fertility, and are involved in plant nutrition and productivity. These fungi facilitate mineral nutrient uptake, particularly phosphorus and nitrogen from soil, producing large networks of fungal hyphae, which spread from colonised roots into the soil [1, 2], receiving plant carbon compounds, in exchange [3]. AMF also provide some ecosystem services, improving plant tolerance to biotic and abiotic stresses, thereby reducing the need for chemical fertiliser and pesticide inputs in agriculture [4]. The symbiosis induces physiological changes in the colonised plants, affecting the fundamental metabolism of the host cells [5, 6], and modulates their secondary metabolism, for example, enhances the activity of the antioxidant enzymatic systems and the biosynthesis of diverse phytochemicals with health-promoting activities [7].

These physiological modifications are, in turn, linked to the activation/repression of several genes, as observed using mRNA sequencing (RNA-Seq) analyses, in a few plant species such as *Medicago truncatula*, *Solanum lycopersicum*, *Solanum tuberosum*, *Glycine max*, *Oryza sativa*, *Lotus japonicus* and *Helianthus annuus* [8–15]. In roots, where AM symbiosis is established, such changes in both plant and fungal transcriptomes were related to mycorrhizal establishment and development, involving post-translational regulation, signalling, transport, hormone metabolism, and biotic and abiotic stresses [15–20].

Compared with pathogenic fungi, many similarities occur in the molecular interaction of plant roots with beneficial microorganisms [21], including the activation of pathogen-related genes, radical oxygen species burst and callose deposition, as part of the AMF transiently induced early defence responses [22]. Moreover, AMF may protect host plants by directly inhibiting deleterious microbe propagation or through induced systemic resistance as observed in of *Solanum lycopersicum*, *Nicotiana tabacum* and *Phaseolus vulgaris* [23].

It is known that besides affecting gene expression, pathogenic fungi and their elicitors, as well as compounds related to plant defence, can activate the expression of transposons, especially retrotransposons (e.g *Nicotiana tabacum* and *Solanum lycopersicum*) [24–31]. In contrast, the influence of AM symbiosis on the activity of transposable elements (TEs) is not known.

Transposable elements (TEs) are DNA sequences, widespread in the nuclear genome of all eukaryotes [32] and potentially moving across the genome. In particular, class I elements (retrotransposons or retroelements, REs) represent the main fraction of the repetitive component of eukaryotic genomes because they transpose by producing RNA intermediates which are copied to cDNA and inserted into different chromosomal sites, leading to their accumulation in the genome. The most frequent REs in plants show two direct long terminal repeats (LTRs) flanking a coding portion. This portion encodes Pol, a polyprotein which includes four enzyme domains, protease, reverse transcriptase (RT), RNaseH and integrase, involved in the mechanism of replication and integration in the chromosomes of the host; and Gag, a protein resembling that of virus-like particles [33]. LTR-REs use these enzymes to transpose. LTR-REs that are missing one or more domains use enzymes produced by other LTR-REs to replicate and move [32]. LTR-REs are most abundant in plant genomes, especially those belonging to the *Copia* and *Gypsy* superfamilies [32], which differ in the position of the integrase domain within the open reading frames [33] and may be distinguished into a number of lineages [34–37].

Transposon dynamics contribute considerably to the evolution of genomes, having primary roles in different genome functions. Besides affecting the genome size [38–40], transposable elements are involved in genome restructuration [41], the generation of new genes by rearrangements of gene segments [42] and affect heterochromatin formation in the nucleus [43]. Finally, transposons can alter the host’s regulatory network and gene expression, at both the structural and epigenetic levels, possibly leading to phenotypic variations [44]. The regulatory role of TEs might have important implications into the switch of the host expression leading to symbiosis.

The sunflower, *Helianthus annuus* (Asteraceae), is widely cultivated and one of the four most important sources of vegetable oil. It has a large genome (about 3.3 Gbp), which has recently been sequenced [45]. Repetitive elements, and especially LTR-REs, represent the vast majority (around 80%) of the genome [46–49]. Natali et al. [47] assembled and annotated the repetitive portion of the sunflower genome, finding that the abundance of *Gypsy* elements exceeds by four-fold that of *Copia* REs. In recent works, different RE lineages to which sunflower LTR-REs belong were identified [48–50]. The *Copia* lineages included *AleI/Retrofit/Hopscotch*, *AleII*, *Angela*, *Bianca*, *Ivana/Oryco*, *Maximus/SIRE* and *TAR/Tork* [32]. Conversely, the most frequent *Gypsy* lineages were *Ogre/Tat* [51], *Athila* [52] and *Chromovirus*, a lineage that is especially abundant in the sunflower genome (it exceeds 30% of the genome [49]) and is generally associated with centromeric regions [51, 53].

In the last 10 years, studies have shown that LTR-REs are transcriptionally active in sunflower [54–56]. The transcription of *Copia* and *Gypsy* REs commonly occurs in different organs (embryos, leaves, roots and flowers) and, reportedly, it is not induced by environmental factors or culture conditions [55]. In the same study, one of 64 individuals of a progeny of a selfed line exhibited the integration of a new element into the genome. Similarly, multiple lineages of *Gypsy* and *Copia* REs were active in natural populations of *H*. *annuus* and *H*. *petiolaris*, and in their natural interspecific hybrids [56].

Concerning the establishment of AMF symbiosis in sunflower, recent investigations performed on sunflower wild accessions, cultivars and inbred lines colonised by *Rhizoglomus irregulare*, demonstrated a relationship between the susceptibility to AMF and the degree of domestication, with wild accessions more susceptible to colonisation than cultivars [57–58].

One of the most studied and widespread arbuscular fungi is *Rhizoglomus irregulare* (formerly known as *Rhizophagus irregulare*), because of its ability to colonise most of plant species [59]. The genome of *R*. *irregulare* is about 153 Mbp and reportedly contains around 36% repeated elements, including LTR-, non-LTR-retrotransposons (also called long interspersed nuclear elements, LINEs) and DNA transposons [60]. *Rhizoglomus irregulare* is said to share multiple identical nuclei in coenocytic hyphae (homokaryon hypothesis) although it has been shown that nuclei can undergo large genomic rearrangements [61], leading to high genome variability, which may affect both genes and repeated elements, in different strains of this fungus [62].

Despite the importance of TEs as regulatory elements, little is known about their involvement in the modulation of host physiology towards symbiosis. This plant-fungus interaction have a considerable importance in exchange of nutrients from the soil, especially phosphate and nitrogen, improving both growth and health of the host plant [3,4]. In addition, the recently sequenced sunflower (*Helianthus annuus*) genome revealed a high distribution of repeats in the genome with an elevate percentage of *Copia* and *Gypsy* retroelements [45]; for these reasons a detailed analysis of retrotransposons activation in mycorrhizal symbiosis between sunflower and *Rhizoglomus irregulare* could bring an important insight into the relationships between two meaningful biological processes such as the plant-fungus interaction and the modulation of transposition events. In this sense, the goal of the present study is to identify differentially expressed TEs which might be involved in sunflower response to AMF colonisation.

An RNA-Seq approach may be exclusively applied to the analysis of LTR-RE expression when the genome sequence or a complete reference library of LTR-REs is available. For example, RNA-Seq has been recently exploited to investigate the expression of TEs in poplar [63]. To analyse the activation and identify the differentially expressed lineages of LTR-REs in sunflower roots during AMF colonisation, we assessed the differential expression of the reverse transcriptase (RT)-encoding domain, by mapping Illumina RNA-Seq libraries onto a database of sunflower and *R*. *irregulare* RE sequences. Moreover, using sunflower genomic resources available in public repositories [45], including Illumina cDNA libraries obtained from roots of plants treated with different hormones and chemicals mimicking stimuli produced by biotic and abiotic stresses, we compared the RT-encoding sequence expression induced by AMF with that induced by these treatments, to infer the factors possibly inducing LTR-RE expression in mycorrhizal sunflower.

## Materials and methods

### Plant material and treatments

Plant materials were the same as described in a previous work [15]. Overall, 40 seeds of *H*. *annuus* (inbred line HA412-HO, USDA Accession Number PI 642777) were germinated on moistened filter paper lied in Petri plates. Then, roots of 1-week-old plantlets were placed on a 90-mm diameter membrane (cellulose acetate–cellulose nitrate, 0.45 μm pore diameter size, MF-Millipore) and covered with a 100 cm^2^ nylon net (41 μm mesh, Millipore). *Rhizoglomus irregulare* (IMA6 strain) spores, mycelium and colonised fine roots, previously obtained from a pot culture soil after wet sieving through a 100-μm sieve, were spread onto nylon net surfaces. Uninoculated control plants received 10 mL of a filtrate, obtained by sieving the mycorrhizal inoculum through a 50 μM pore diameter sieve and then through Whatman paper no. 1 (Whatman International Ltd, Maidstone, Kent, UK), to ensure a common AMF-associated microflora to all treatments. For each inoculated and non-inoculated plantlet, another 90 mm membrane was placed on the nylon net, and then the whole system was transferred to sterile 150-mm Petri plates containing steam-sterilised quartz grit. Plates were sealed with Parafilm M, and the lower half was wrapped in aluminium foil. Then, all plantlets were transferred to a growth chamber at 24°C. Half-strength Hoagland’s solution (6 ml) was provided weekly, and progression of fungal colonisation was assessed by succinate dehydrogenase activity and trypan blue staining [64, 65], monitored every 48 h on three plantlets. The gridline intersect method [66] was used to determine the percentage of AM fungal root colonisation.

### RNA isolation extraction, sequencing and mapping procedures

Whole roots of mycorrhizal and control plantlets harvested after culture for 4 and 16 days, respectively, were ground in liquid nitrogen, and total RNA was isolated using the Logemann procedure [67].

DNaseI (Roche, Basel, Switzerland) was used to purify the RNA from genomic DNA, followed by phenol–chloroform extraction, and precipitation, using standard procedures. The overall RNA quality was evaluated by Bioanalyser 2100 (Agilent Technologies, Santa Clara, CA).

Messenger RNA (mRNA) was converted to cDNA and 12 cDNA libraries (three replicates of mycorrhizal and control root systems at 4 and 16 days of culture) were constructed by using a TruSeq RNA sample preparation kit (Illumina, Inc., San Diego, CA, USA), according to the manufacturer’s protocol.

Single-read sequences of 100 bp length were obtained by Illumina HiSeq 2000. FastQC (v.0.11.3) was used to check the global reads quality, and Trimmomatic [68] yielded high-quality reads trimmed with the following parameters: crop = 95, head crop = 10, minimum length = 85.

In order to exclude any ribosomal RNA (rRNA) traces from the libraries, reads were aligned against sunflower rRNA sequences downloaded from the NCBI repository, with the following criteria: length fraction = 0.5, similarity fraction = 0.8.

### Production of a database of reverse transcriptase (RT)-encoding sequences of *Helianthus annuus* and *Rhizoglomus irregulare*

Reverse transcriptase (RT)-encoding sequences from LTR-retrotransposons of *H*. *annuus* were identified in a sequence set, representing a whole-genome set of assembled sequences [47]. Briefly, Illumina reads were obtained from genomic DNA of the HA412-HO line (the same used for establishment of symbiosis) and assembled using various procedures [47].This sequence set was analysed by using the RepeatExplorer [69] protein domain search tool. Searches were performed against the Repbase database of RT protein domains derived from mobile elements in plant and included in the RepeatExplorer tool, and RT sequences were retained using the following parameters: 60% minimum similarity, 40% minimum identity; proportion of the hit length from the length of the database sequence = 0.3; maximum allowed frameshifts = 3.

cDNA sequences obtained from inoculated and control roots of sunflower (see above) were mapped to the database of RT sequences using CLCBio Genomics Workbench version 9.5.3 (CLCBio, Aarhus, Denmark), with the following parameters: length fraction = 0.7, similarity fraction = 0.9, mismatch penalties = 1, gap penalties = 1.

*Rhizoglomus irregulare* genomic Illumina reads were downloaded from the Sequence Read Archive (SRA) public repository (accession number SRR2727640) and trimmed by Trimmomatic (SLIDINGWINDOW:4:20, MINLEN:125 [68]. Then, a randomised sample of 2,000,000 paired-end reads was assembled with RepeatExplorer [67] and compared with the Repbase database of fungi repeated elements. Reverse Transcriptase sequences with a 30% minimum identity, 60% minimum similarity, 150 bp minimum length and a maximum of three frameshifts were retained. Such parameters were less stringent than those used for *H*. *annuus*, as suggested by RepeatExplorer instructions for non-higher plant species.

Sequences obtained from the assembly were then retrieved on the genomes of six strains of *R*. *irregulare* [62], using the BLASTN suite (max_target_seqs = 1; with all default parameters).

### Abundance estimation of reverse transcriptase (RT) sequences in the *Helianthus annuus* genome

The abundance of each RT-encoding sequence in the genome of *H*. *annuus* line HA412-HO was estimated by mapping an Illumina set of 9,871,724 Illumina reads (cleaned from organellar sequences), trimmed to 90 nt in length (coverage 0.24×) to the isolated RT sequences (Bioproject archive accession number PRJNA64989). Mapping was conducted using CLCBio Genomics Workbench version 9.5.3. This tool distributes multireads randomly; however, the number of multireads is generally very low, and the number of mapped reads to a single sequence is an indication of its redundancy. Mapping was performed using mismatch cost = 1, deletion cost = 1, insertion cost = 1, similarity fraction = 0.9, and length fraction = 0.7.

### Differential expression analysis

The expression level of RT-encoding sequences was calculated both as reads per kilobase per million reads mapped (RPKM) [70] and as mapped reads per million. Mapping was performed separately on *H*. *annuus* and *R*. *irregulare* RT sequence sets, using the following parameters: length fraction = 0.7, similarity fraction = 0.9, mismatch penalties = 1, gap penalties = 1. Besides unique reads, reads that occurred up to ten times were also included in the mapping analysis, because this strategy correctly estimates the expression of paralogous RT sequences [70]. Reverse transcriptase (RT) expression was filtered to ensure at least one mapped read per million, in at least one library.

Raw counts derived from the CLC aligner were analysed using Baggerley’s statistical test, i.e. a proportion based comparison on t-test weighted by beta-distribution [71]. A pairwise comparison test was performed between mycorrhizal (M) and control libraries (C) after culture with AMF for 4 and 16 days, respectively (C4 vs. M4 and C16 vs. M16). The resulting *P*-values were corrected for the false discovery rate (FDR) [72], and significant RT sequences were identified (*P*<0.05, FDR-corrected).

### Analysis of reverse transcriptase (RT) sequence expression in hormonal and stress treatments

In order to compare the expression of RT-encoding sequences induced by AMF with that induced by other treatments, 10 Illumina cDNA libraries from sunflower roots (inbred line HanXRQ), each treated with different hormones and chemicals, mimicking stimuli produced during biotic and abiotic stresses [45], were downloaded from the NCBI SRA (accession number SRP092742). Libraries were built from roots of plants treated with auxin, ethylene, gibberellic acid, salicylic acid, kinetin, abscisic acid, strigolactones, brassinosteroid, polyethylene glycol and NaCl, respectively. One library per treatment and seven corresponding control libraries were available [45].

The quality of the reads was checked using FastQC (v. 0.11.3), and overall quality was improved by trimming the reads using Trimmomatic [68], and removing Illumina adapter (Illuminaclip, Headcrop:10, Crop:56). Ribosomal reads were removed, and mapping was performed versus RT sequences using the CLC Genomics Workbench (v. 9.5.3, same parameters described above). Reverse transcriptase (RT)-encoding sequences showing more than one mapped read per million for each treatment were considered as expressed. Statistical analyses of differential expression between treatment and control libraries were done using Baggerley’s statistical test [71].

## Results

### Collection of reverse transcriptase (RT)-encoding sequences

A genome-wide analysis was conducted to identify RT-encoding sequences from the whole-genome assembly of the HA412-HO inbred line [47]. This assembly was composed of 283,800 scaffolds or contigs. These were scored for the occurrence of RT-encoding sequences of LTR-REs, using the domain search tool of RepeatExplorer [67]. This analysis was limited to LTR-retrotransposons because the frequency of other autonomous retrotransposons (namely, LINEs) in the sunflower genome is negligible [47].

**Fig 1:**
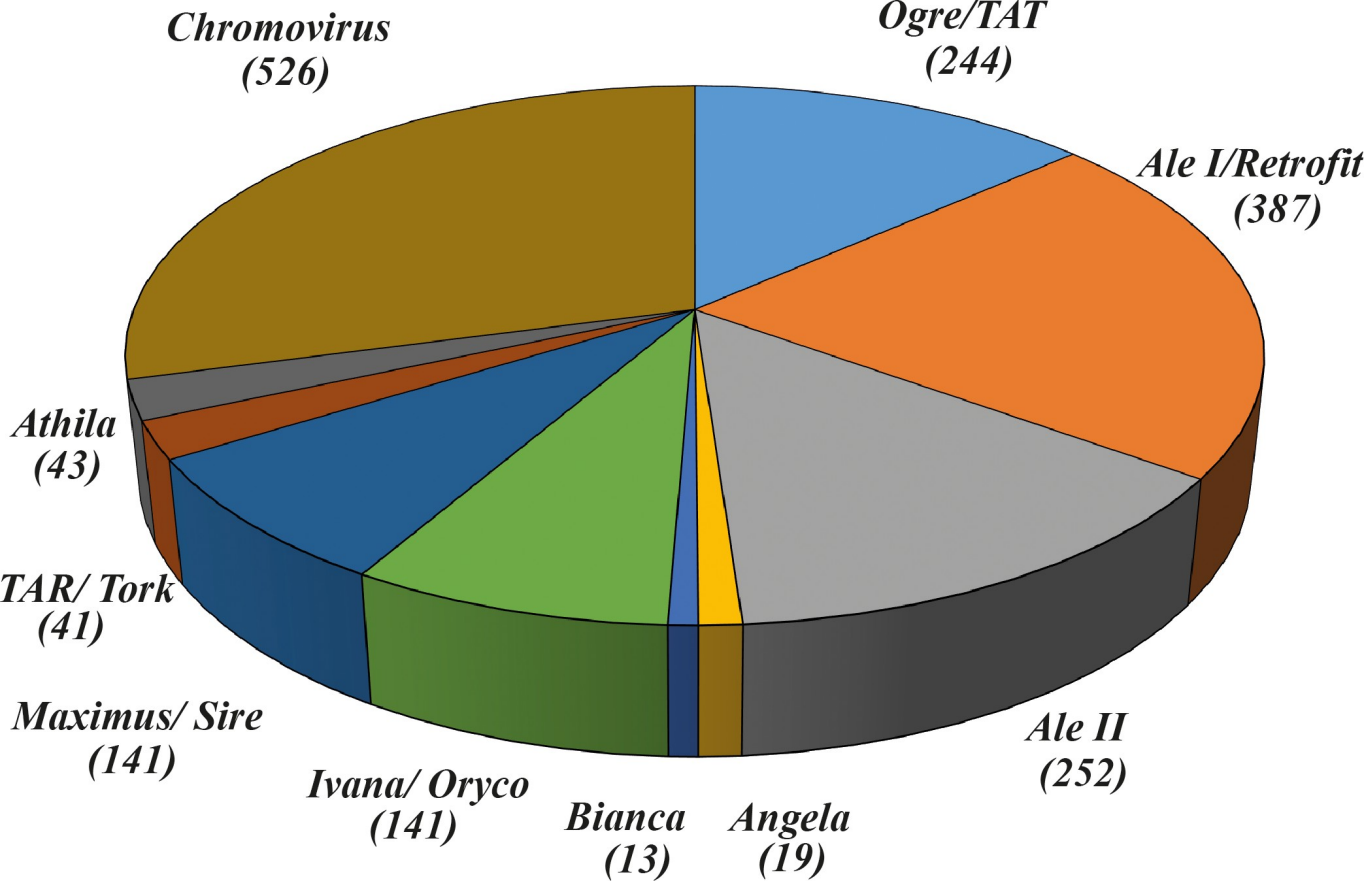
Composition of the library of long terminal repeat-retrotransposon reverse transcriptase sequences of sunflower.

Sequences showing more than 90% similarity were discarded, and a total of 1,807 unique RT sequences were retained, belonging to seven lineages of the *Copia* superfamily and three lineages of the *Gypsy* superfamily. The composition of this RT sequence set is reported in Fig 1.

**Fig 2:**
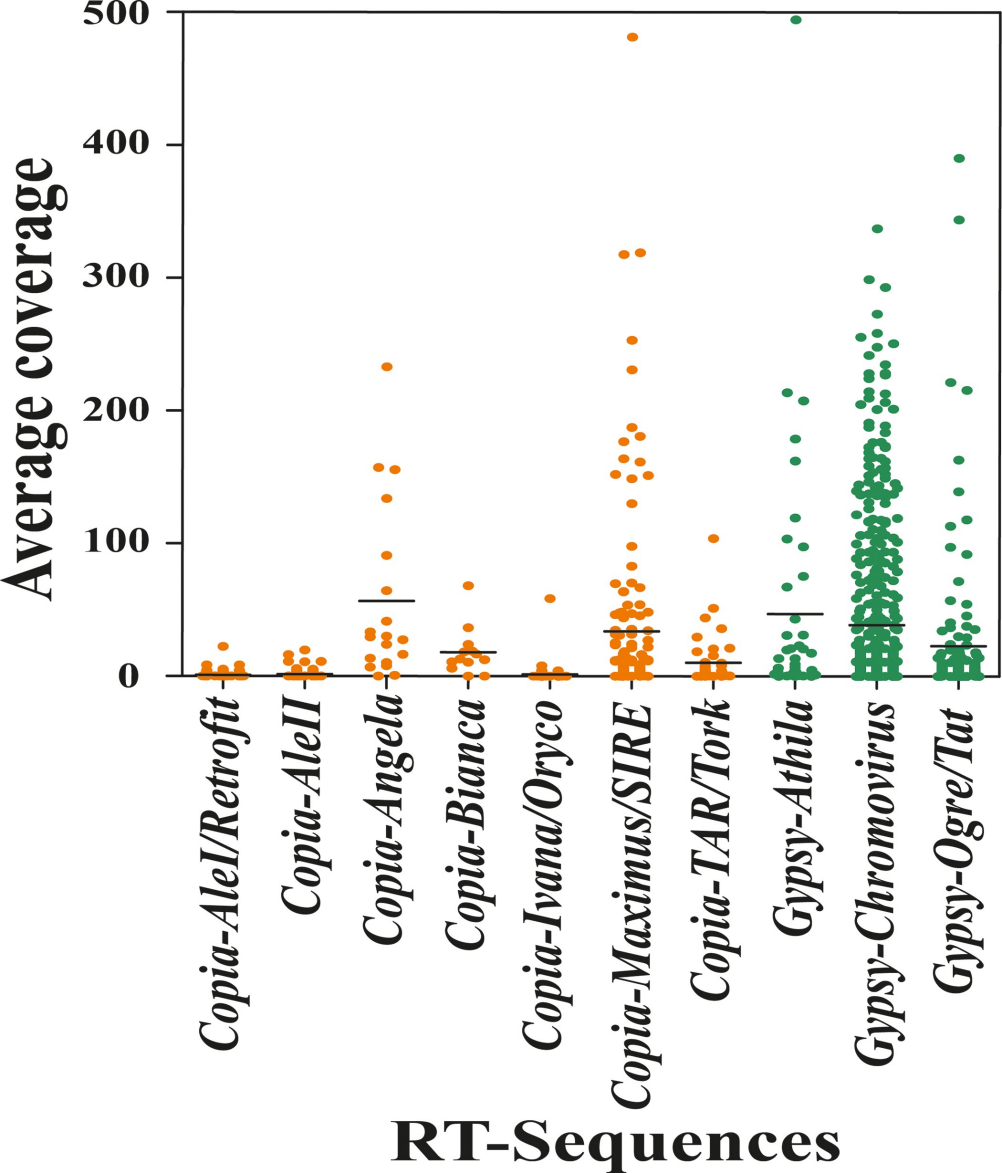
Average coverage of each of 1,807 unique reverse transcriptase (RT)-encoding sequences identified in the sunflower genomes, separated according to the long terminal repeat-retrotransposon lineage to which the RT sequence belongs. Bar represents the mean average coverage of each lineage.

The abundance of each RT was measured by mapping the Illumina reads onto the RT sequence set. The average coverage of aligned reads (the sum of the bases of the aligned part of all the reads divided by the length of the reference sequence) of each sequence is reported in Fig 2. As expected, the most abundant sequences belonged to the *Chromovirus* and *Ogre/Tat* lineages of the *Gypsy* superfamily [46–49] (Fig 2).

For *R*. *irregulare*, Illumina reads were assembled with RepeatExplorer and 31 clusters of contigs containing sequences of *Copia*, *Gypsy* and LINE retrotransposons, each representing at least 1% of the fungal genome, were collected, totalling 814 sequences.

All the sequences, except 14, matched to the *R*. *irregulare* genomes [62]. After filtering for RT domains, 33 sequences were retrieved. Most RT sequences belonged to LINEs (66%) and *Gypsy* LTR-REs (34%). No RT sequences belonging to *Copia* elements were identified. Nonetheless, it is to be considered that the large intraspecific variations in sequence and structure of *R*. *irregulare* genome could have made difficult matching assembled sequences to the genomes.

### Expression of reverse transcriptase (RT)-encoding sequences in sunflower mycorrhizal roots

*Rhizoglomus irregulare-*inoculated roots showed the first signs of colonisation after culture for 4 days while at 16 days, the percentage of colonised root length was about 50%. Control roots did not show any fungal structure throughout the experiment. Based on these data, RNA-Seq analyses were performed on mycorrhizal and control roots harvested at 4 (M4 and C4, respectively) and 16 (M16 and C16) days of culture [15]. The total number of high-quality reads for each of the 12 libraries ranged from 17.9 to 62.8 million. After aligning high-quality reads to the sunflower RT-encoding sequence set, the percentage of mapped reads of each sample to the sequence set ranged from 0.02% to 0.08%.

Overall, only 46 out of 1,807 sequences (2.55%) were transcribed (i.e., they showed more than one mapped read × million) in at least one Illumina mycorrhizal or control library. Almost all (43/46) belonged to the *Copia* superfamily, and specifically to the lineages *AleII* (22 elements), *Ivana/Oryco* (6), *Maximus/SIRE* (5), *Angela* (4), *Bianca* (2), *TAR/Tork* (2) and *AleI/Retrofit* (1). The three expressed RT sequences belonging to the *Gypsy* superfamily were all of the *Chromovirus* lineage.

The expression level (in mapped reads × million) is reported in Table 1. The mean number of reads matching RT sequences was very low compared to the number of reads mapping to genes (S1 Table). It ranged from 11.80 to 21.68 in the four samples, and the maximum number of mapped reads per million ranged from 188.94 to 320.05. These maximum values belonged to one element of the *Copia* lineage *AleII*.

**Table 1:** Number of mapped reads onto 46 reverse transcriptase (RT)-encoding sequences expressed in *Helianthus annuus*, at 4 or 16 days after arbuscular mycorrhizal fungal inoculation (M4 and M16) and/or in the respective controls (C4 and C16). For each sample, the maximum and the minimum number of reads mapping to single RT sequences are also reported. Values are the means of mapping reads per million, using triplicates per sample.

Sample	Mean nr. of reads per million	Minimum nr. of reads per million	Maximum nr. of reads per million
C4	13.66	0.65	188.95
M4	21.68	0.63	320.05
C16	14.10	0.56	228.38
M16	11.80	0.70	212.14

Interestingly, the mean number of reads matching RT sequences was similar in C4, C16 and M16 samples while it increased in M4; the number of reads matching RT sequences dropped from 21.68 in M4, to 11.80 in M16, i.e., it decreased during the colonisation process (Table 1).

**Fig 3:**
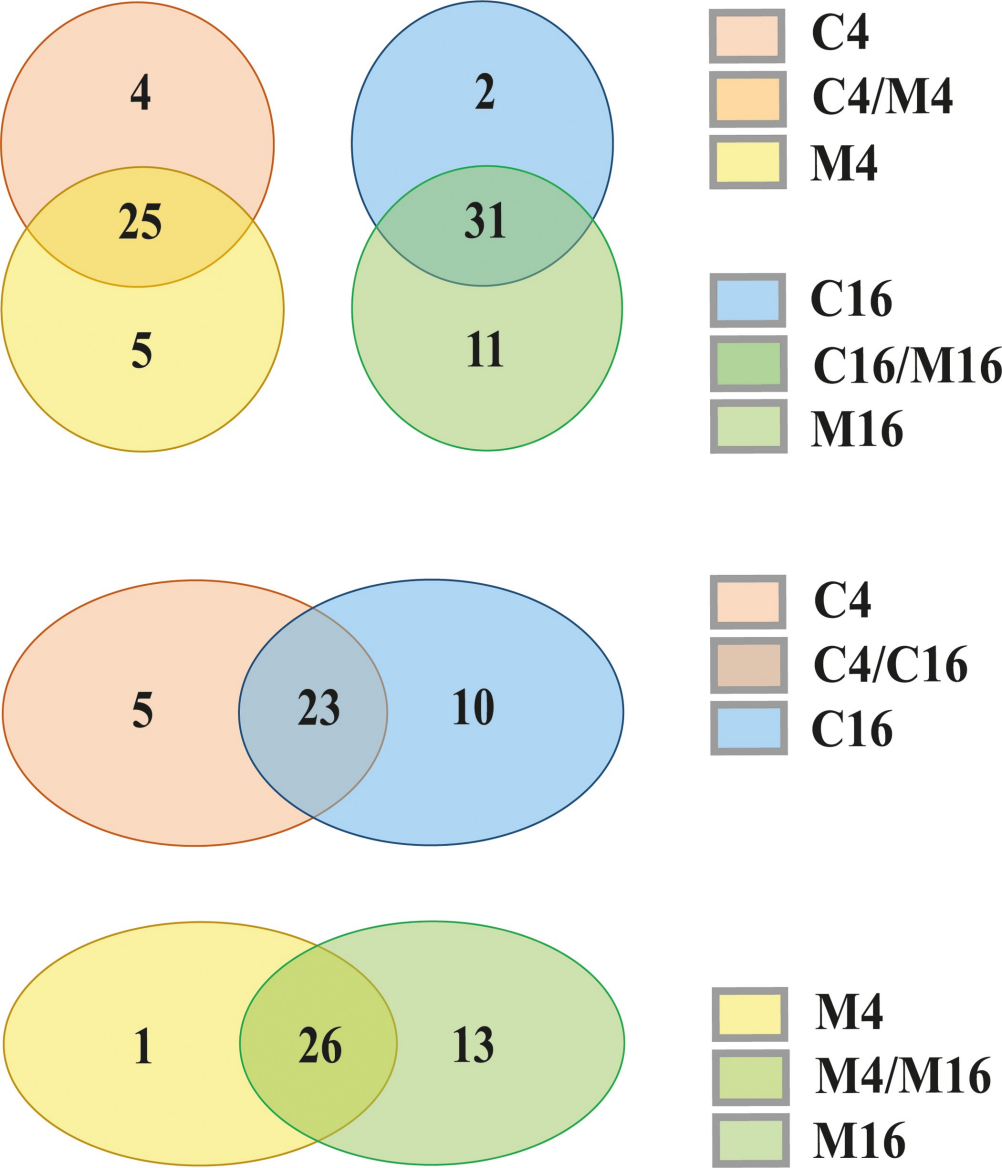
Number of expressed reverse transcriptase sequences (more than one mapped read × million in at least one library) in *Helianthus annuus* roots at 4 and 16 days after arbuscular mycorrhizal fungal inoculation (M: mycorrhizal roots; C: control roots).

By comparing the transcription values in mycorrhizal with control plants at 4 and 16 days, it can be observed that most elements were expressed in both mycorrhizal and control samples (Fig 3). Similarly, comparing early with late treatments or early with late controls, the majority of RT sequences were expressed throughout the experiment (Fig 3). Overall, 22 out of 46 expressed RT sequences were present in each of the four samples (C4, C16, M4 and M16), suggesting that the corresponding elements were constitutively expressed (even if at a low rate). Fourteen out of 22 sequences belonged to the *AleII* lineage of the *Copia* superfamily.

**Fig 4:**
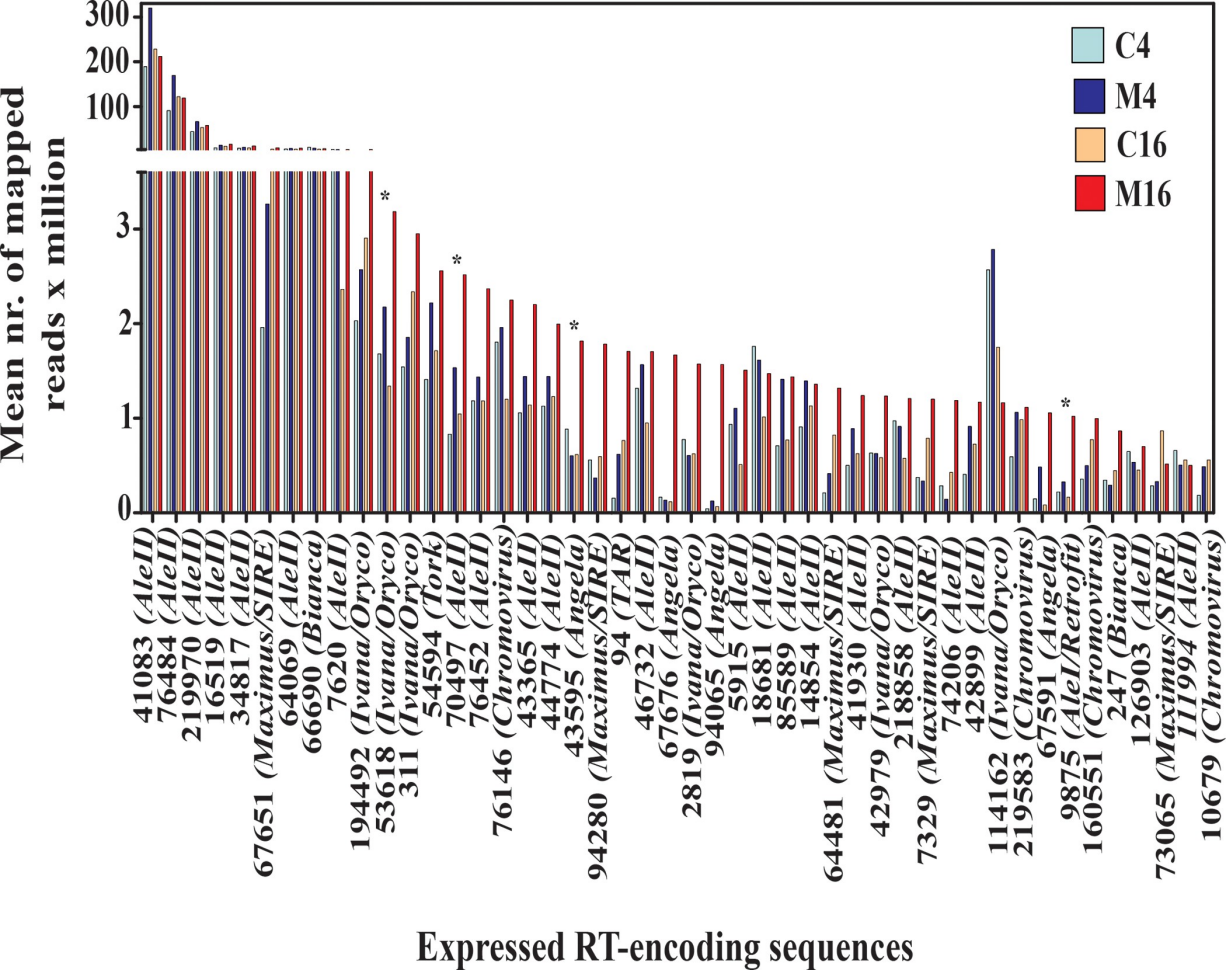
Mapped reads per million of 46 expressed (more than one mapped read x million in at least one cDNA Illumina library) reverse transcriptase (RT)-encoding sequences in *Helianthus annuus* mycorrhizal (M) and control roots (C) after culture for 4 and 16 days. Asterisks indicate significant over-expressed RT sequences (at day 16).

The differential expression of the 46 expressed RT-encoding sequences was analysed (Fig 4). No RT sequence was differentially expressed after AMF colonisation for 4 days, compared with the respective controls. After AMF colonisation for 16 days, only four RT-encoding sequences were significantly over-expressed in mycorrhizal roots (Fig 4). No RT sequence was under-expressed during the experiment. The four differentially expressed RT sequences showed a low average number of mapped reads per million values (from 0.166 to 3.182, compared to the mapped reads per millions of housekeeping or AMF symbiosis-induced genes, S1 Table) and all belonged to the *Copia* superfamily, one each for the *AleI/Retrofit*, *AleII*, *Angela* and *Ivana/Oryco* lineages (Fig 4).

Since, in certain cases, the occurrence of RE sequences in cDNA libraries can be related to genomic DNA contamination, we analysed the relationship between the RPKM value of expressed RT sequences in the M16 sample and their abundance in the genome, as indicated by the average coverage (S1 Fig). The correlation coefficient was very low (*r* = 0.1), so contamination by genomic DNA in the cDNA libraries could be largely ruled out. Moreover, the most abundant RT sequences were slightly expressed, and, correspondingly, the most expressed RTs were poorly represented in the genome of Ha412-HO inbred line, except one RT sequence with an average coverage of over 400 and about five mapped reads per million. Considering the four differentially-transcribed RT sequences, genomic DNA contamination of cDNA libraries could be excluded (S1 Fig).

In order to analyse the expression of fungal retrotransposon during colonisation, reads from mycorrhizal libraries at 4 and 16 days of colonisation were mapped to the 33 RT-encoding sequences of LINE and *Gypsy* from the *R*. *irregulare* genome, whereas, no read aligned to the fungal RT sequences. This finding indicated that either fungal retrotransposons are not expressed during symbiosis or that the coverage of fungal reads was too low to estimate the RT expression rate if any.

### Comparative analysis of reverse transcriptase (RT) sequence expression

**Fig 5:**
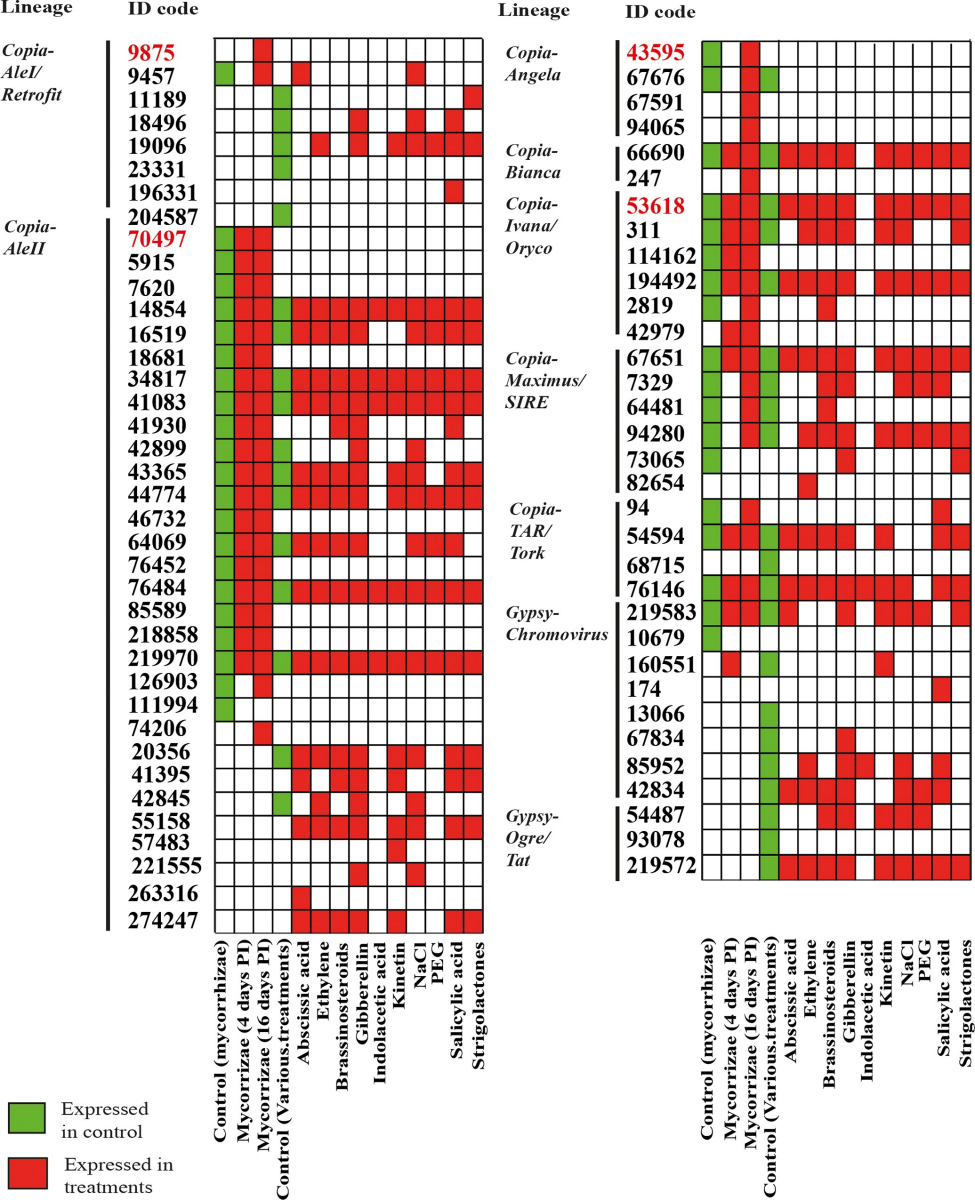
Expression of reverse transcriptase (RT)-encoding sequences after different treatments of *Helianthus annuus* plants. A sequence was considered as expressed when matching more than one read per million in mapping cDNA libraries to the set of sunflower RT sequences. Libraries from cDNA of mycorrhizal roots and their controls were prepared during this study; the other libraries [45] were collected from the public database (see the materials and methods section). Green cells refer to the average expression of control libraries. PI = Post Inoculation.

**Fig 6:**
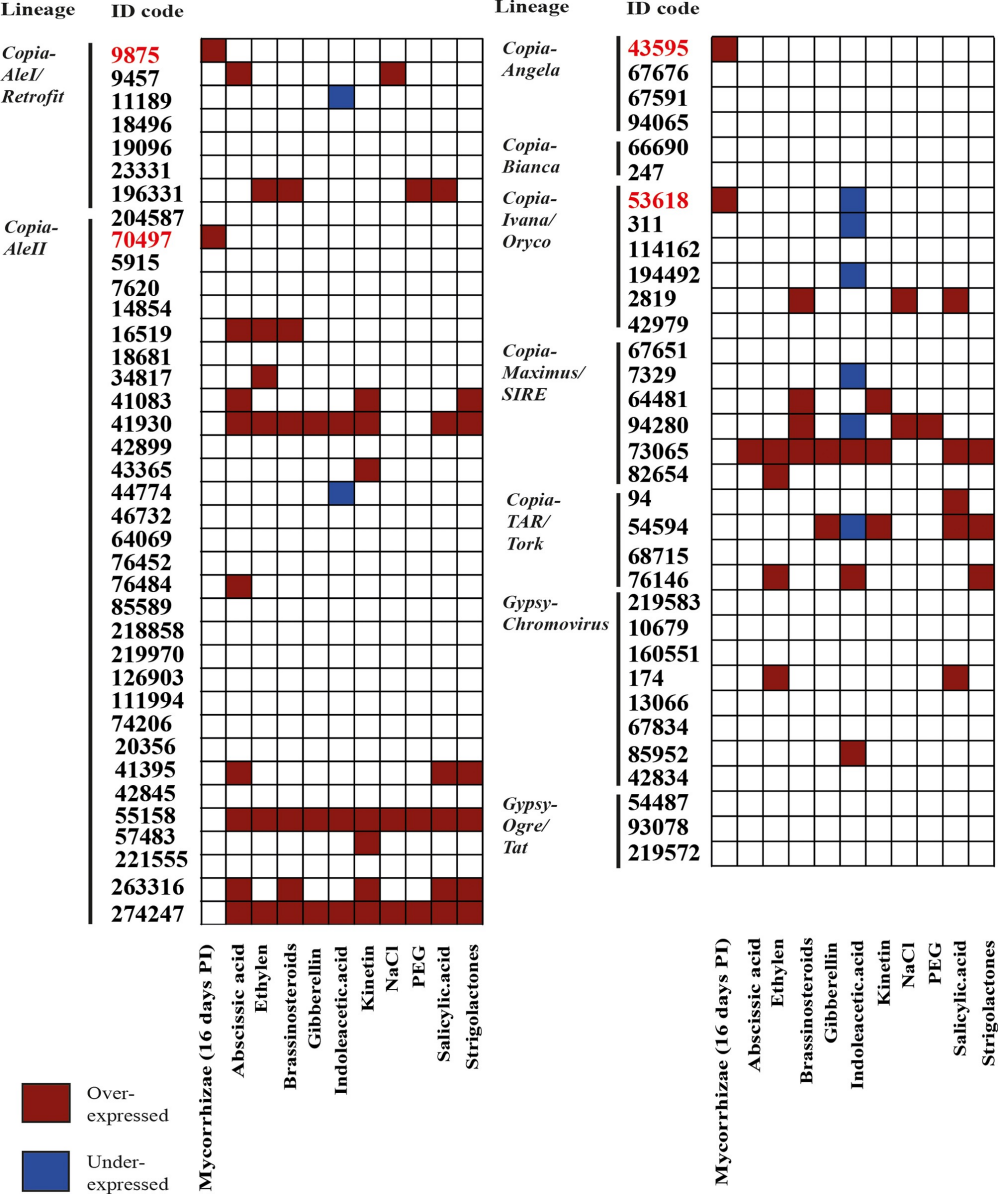
Differential expression of reverse transcriptase (RT)-encoding sequences after different treatments of *Helianthus annuus* plants. Analysis was performed only on expressed sequences. Libraries from cDNA of mycorrhizal roots and their controls were prepared during this study; the other libraries [45] were collected from the public database (see the materials and methods section).

Reverse transcriptase (RT) sequence expression was estimated by mapping onto the same RT sequence library as mentioned above, 10 Illumina cDNA libraries publicly available [45], obtained from roots treated with abscisic acid, ethylene, brassinosteroids, gibberellic acid, indole-acetic acid, kinetin, NaCl, polyethylene glycol, salicylic acid and strigolactones, respectively. Although the ten cDNA libraries were obtained from the HanXRQ genotype rather than Ha412-HO, all RT sequences occurred in both genotypes (data not shown), indicating that the corresponding LTR-RE families are shared between the two genotypes. The results of this comparative analysis are reported in Fig 5 and Fig 6, in which the differential expression in controls and M4 and M16 roots is illustrated.

A large number of RT sequences were expressed in many treatments, confirming the widespread transcription of LTR-REs in sunflower [55, 56]. Thirty RT sequences expressed in mycorrhizal roots were also expressed in at least one of the other treatments. Sixteen RT sequences were expressed only in the specimens used in this study (mycorrhizal and/or control treatments, Fig 5).

Concerning differentially expressed RT sequences (Fig 6), all treatments determined over-expression of RT sequences, except indole-acetic acid, which induced under-expression of some RT sequences. The four significantly over-expressed RT sequences induced by mycorrhizal symbiosis were not activated by any other treatment, suggesting that the mechanism of RE induction by AMF is rather specific and not amenable to any of the chemical or hormonal treatments tested here. Instead, two of the analysed RT sequences were induced by all treatments, except mycorrhization.

## Discussion

McClintock [73] addressed the activation of TEs as a possibility for the genome to cope with changes in the environment in which a species lives, for example, exposure to biotic and abiotic stresses. Indeed, active mobile elements can produce new genetic variability, increasing the possibility to overcome changing environments, with a significant impact on the evolution of the species.

It is known that pathogenic fungi can induce the activation of REs of the host plant [24–31]. To our knowledge, the effect of AMF root colonisation on REs of the genome of the host species has not been described so far, using the newly available sequencing technologies.

To infer the potential impact of AMF colonisation on TE activation, we analysed the expression of RT sequences during mycorrhization in sunflower. Finding an expressed RT sequence does not ensure that the RT belongs to a functional retrotransposon and that the complete retrotransposon is transcribed. However, given the generally low level of retrotransposon transcription and the length of these elements, an analysis of the expression of a complete element would need cDNA sequence coverage largely exceeding those obtained in current RNA-Seq experiments.

The observed RT sequence expression rate in mycorrhizal roots was on average very low compared with gene sequences, and the vast majority of the RT sequences included in the sunflower dataset was not expressed. The highest total number of cDNA reads matching RT sequences was found in M4, suggesting that at the onset of AMF colonisation, the host reacts, thereby activating REs. Then, with the exception of a few elements, RT transcription values was even lower than those found in controls.

In total, only 46 of 1,807 sequences were transcribed in mycorrhizal or control roots. Among the transcribed RT sequences, a large number were also expressed in hormonal or chemical treatments, suggesting that these RTs belong to LTR-REs, which are regularly active in sunflower roots, even if at low rates. Consistent with this finding, constitutive expression of LTR-REs was previously demonstrated in sunflower and related species [54–56].

In comparison, the induction of specific RTs by AMF colonisation is even rarer. Statistical analysis showed that no RT sequence was differentially expressed at day 4, and only four RT sequences were over-expressed at day 16 after mycorrhizal inoculation. Moreover, although not supported by statistical treatment of the data, it could be observed that five and eleven RT sequences were transcribed in M4 and M16, respectively, without being expressed in their respective controls. In all these cases, it is possible to hypothesise the occurrence, in the related LTR-REs, of promoter sequences specifically activated during mycorrhization.

In general, activation of REs can be associated with a reshaping of the gene regulation network in response to both abiotic and biotic stress. Probably these four REs are specifically regulated as are the genes induced by symbiosis. In the case that such expression activation was followed by retrotranscription and insertion in the genome, it should be assumed that symbiosis can induce genomic changes in the host. Obviously, such possibility is limited by the low number—only four—of REs potentially activated after mycorrhization.

Transposon activity is generally repressed, mostly based on RNA interference, a process that is mediated by small RNAs originating from many different precursors. In general, double-stranded RNAs (dsRNAs) are produced by the host and trigger retrotransposon silencing by chromatin remodelling, RNA-directed DNA methylation and post-transcriptional degradation of RE RNAs [44, 74, 75]. For example, in the yeast *Schizosaccharomyces pombe*, small RNAs maintaining heterochromatin structure through histone methylation are produced following a basal level of transcription of centromeric DNA repeats [76].

Although *Gypsy* LTR-REs are by far more abundant than *Copia* ones in the sunflower genome [77], most expressed RT sequences during mycorrhizal colonisation belonged to LTR-REs of the *Copia* superfamily, and especially to the *Copia* lineage *AleII*. The four RT sequences differentially expressed at M16 also belonged to four different lineages of the *Copia* superfamily. The higher expression of *Copia* elements compared with *Gypsy* ones can be related to the low redundancy of LTR-REs of this superfamily in the sunflower genome. Indeed, it is presumable that low-copy elements escape RNA interference relatively more easily because their past activity is too low to provide the host with enough transcripts to produce specific dsRNAs, thereby reducing the efficiency of silencing mechanisms. Accordingly, in plants, new insertion events were shown only for a few elements, all belonging to the *Copia* superfamily, Tnt1, Tto1 and Tos17, which are present in a relatively low copy number (<1,000) per haploid genome [78].

Concerning the retrotransposons of *R*. *irregulare*, we confirmed the occurrence of LINE and *Gypsy* RT-encoding sequences in the genome, as already reported [60]. No RT expression was found during mycorrhizal colonisation. However, it was not possible to affirm whether fungal retrotransposon expression did not occur during symbiosis or the coverage of fungal reads in our mycorrhizal libraries was too low to detect transcription of sequences that are generally barely expressed.

Sunflower mycorrhizal over-expressed RT sequences were not induced by any hormonal or chemical treatments mimicking abiotic or biotic stresses. Moreover, compared with other treatments analysed in this study, AMF colonisation was the treatment that induced the lowest number of RT sequences: four sequences were over-expressed after AMF colonisation while the other treatments induced from 5 to 15 RT-encoding sequences. These data indicate that mechanisms inducing transcription of RT sequences (and, presumably, of LTR-RE families) by AMF colonisation are highly specific. However, as noted above, the transcription of a retrotransposon does not necessarily imply that the retrotransposon is active and able to transpose. Transcripts should be reverse-transcribed to cDNA, which should be then integrated into the genome. In addition, transcripts might be inactivated at the post-transcriptional level, as shown in other species, including humans [44].

In conclusion, the current work showed the activation of RT-encoding sequences in *H*. *annuus* roots during mycorrhizal colonisation and compared the expression of these elements with other abiotic and biotic stresses, including pathogenic fungi. It could be deduced that mechanisms of retrotransposon activation in the mycorrhizal symbiosis are specific and a few specific retrotransposons are uniquely activated during the mycorrhizal interaction. Further studies will focus on the activation of REs during the various stages of mycorrhizal establishment in different genotypes, to confirm such specificity and disclose its molecular bases, thereby clarifying the role of retrotransposons during the symbiotic interaction.

## Supporting information

S1 Table Expression values of four house-keeping genes of sunflower roots during arbuscular mycorrhizal infection colonization. (DOC)

S1 Fig Relationship between expression and average coverage of each of the 46 RT sequences expressed in mycorrhizal roots at 16 d after inoculation. (DOC)

## References

[1] Battini F, Grønlund M, Agnolucci M, Giovannetti M, Jakobsen I. Facilitation of phosphorus uptake in maize plants by mycorrhizosphere bacteria. Sci Rep. 2017; 7:4686 10.1038/s41598-017-04959-0 28680077 PMC5498536

[2] Pepe A, Sbrana C, Ferrol N, Giovannetti M. An in vivo whole-plant experimental system for the analysis of gene expression in extraradical mycorrhizal mycelium. Mycorrhiza. 2017; 572:1–10. 10.1007/s00572-017-0779-7 28573458

[3] Smith SE, Read DJ. Mycorrhizal symbiosis. Cambridge, UK: Academic Press 2008.

[4] Gianinazzi S, Gollotte A, Binet MN, van Tuinen D, Redecker D, Wipf D. Agroecology: the key role of arbuscular mycorrhizas in ecosystem services. Mycorrhiza. 2010; 20:519–530. 10.1007/s00572-010-0333-3 20697748

[5] Lohse S, Schliemann W, Ammer C, Kopka J, Strack D, Fester T. Organization and metabolism of plastids and mitochondria in arbuscular mycorrhizal roots of *Medicago truncatula*. Plant Physiol. 2005; 139:329–340. 10.1104/pp.105.061457 16126866 PMC1203382

[6] Araim G, Saleem A, Arnason JT, Charest C. Root colonization by an arbuscular mycorrhizal (AM) fungus increases growth and secondary metabolism of purple coneflower, *Echinacea purpurea* (L.) *Moench*. J Agr Food Chem. 2009; 57:2255–2258. 19239187 10.1021/jf803173x

[7] Avio L, Turrini A, Giovannetti M, Sbrana C. Designing the ideotype mycorrhizal symbionts for the production of healthy food. Front Plant Sci. 2018; 9:1089 10.3389/fpls.2018.01089 30154803 PMC6102486

[8] Grunwald U, Nyamsuren O, Tamasloukht M, Lapopin L, Becker A, Mann P, et al Identification of mycorrhiza-regulated genes with arbuscule development-related expression profile. Plant Mol Biol. 2004; 55:553–566. 10.1007/s11103-004-1303-y 15604700

[9] Fiorilli V, Catoni M, Miozzi L, Novero M, Accotto GP, Lanfranco L. Global and cell-type gene expression profiles in tomato plants colonized by an arbuscular mycorrhizal fungus. New Phytol. 2009; 184:975–987. 10.1111/j.1469-8137.2009.03031.x 19765230

[10] Gallou A, Declerck S, Cranenbrouck S. Transcriptional regulation of defence genes and involvement of the WRKY transcription factor in arbuscular mycorrhizal potato root colonization. Funct Integr Genomics. 2012; 12:183–198. 10.1007/s10142-011-0241-4 21811781

[11] Hogekamp C, Küster H. A roadmap of cell-type specific gene expression during sequential stages of the arbuscular mycorrhiza symbiosis. BMC Genomics. 2013; 14:306 10.1186/1471-2164-14-306 23647797 PMC3667144

[12] Schaarschmidt S, Gresshoff PM, Hause B. Analyzing the soybean transcriptome during autoregulation of mycorrhization identifies the transcription factors GmNF-YA1a/b as positive regulators of arbuscular mycorrhization. Genome Biol. 2013; 14:R62 10.1186/gb-2013-14-6-r62 23777981 PMC3706930

[13] Fiorilli V, Vallino M, Biselli C, Faccio A, Bagnaresi P, Bonfante P. Host and non-host roots in rice: cellular and molecular approaches reveal differential responses to arbuscular mycorrhizal fungi. Front Plant Sci. 2015; 6:636 10.3389/fpls.2015.00636 26322072 PMC4534827

[14] Handa Y, Nishide H, Takeda N, Suzuki Y, Kawaguchi M, Saito K. RNA-seq transcriptional profiling of an arbuscular mycorrhiza provides insights into regulated and coordinated gene expression in *Lotus japonicus* and *Rhizophagus irregularis*. Plant Cell Physiol. 2015; 8:1490–1511. 10.1093/pcp/pcv071 26009592

[15] Vangelisti A, Natali L, Bernardi R, Sbrana C, Turrini A, Hassani-Pak K, et al Transcriptome changes induced by arbuscular mycorrhizal fungi in sunflower (*Helianthus annuus* L.) roots. Sci Rep. 2018; 8:4 10.1038/s41598-017-18445-0 29311719 PMC5758643

[16] Brechenmacher L, Weidmann S, Van Tuinen D, Chatagnier O, Gianinazzi S, Franken P, Gianinazzi-Pearson V. Expression profiling of up-regulated plant and fungal genes in early and late stages of *Medicago truncatula*-*Glomus mosseae* interactions. Mycorrhiza. 2004; 14:253–262. 10.1007/s00572-003-0263-4 13680319

[17] Gomez SK, Javot H, Deewatthanawong P, Torres-Jerez I, Tang Y, Blancaflor EB, et al *Medicago truncatula* and *Glomus intraradices* gene expression in cortical cells harboring arbuscules in the arbuscular mycorrhizal symbiosis. BMC Plant Biol. 2009; 9:10 10.1186/1471-2229-9-10 19161626 PMC2649119

[18] Hogekamp C, Arndt D, Pereira PA, Becker JD, Hohnjec N, Küster H. Laser micro dissection unravels cell-type-specific transcription in arbuscular mycorrhizal roots, including CAAT-Box transcription factor gene expression correlating with fungal contact and spread. Plant Physiol. 2011; 157:2023–2043. 10.1104/pp.111.186635 22034628 PMC3327204

[19] Gaude N, Schulze WX, Franken P, Krajinski F. Cell type-specific protein and transcription profiles implicate periarbuscular membrane synthesis as an important carbon sink in the mycorrhizal symbiosis. Plant Signal Behavior. 2012; 7:461–464. 10.4161/psb.19650 PMC3419033 22499167

[20] Shu B, Li W, Liu L, Wei Y, Shi S. Transcriptomes of arbuscular mycorrhizal fungi and litchi host interaction after tree girdling. Front Microbiol. 2016; 7:408 10.3389/fmicb.2016.00408 27065972 PMC4811939

[21] De Coninck B, Timmermans P, Vos C, Cammue BPA, Kazan K. What lies beneath: below ground defense strategies in plants. Trends Plant Sci. 2015; 20:91–101. 10.1016/j.tplants.2014.09.007 25307784

[22] Cameron DD, Neal AL, van Wees SC, Ton J. Mycorrhiza-induced resistance: more than the sum of its parts? Trends Plant Sci. 2013; 18:539–545. 10.1016/j.tplants.2013.06.004 23871659 PMC4194313

[23] Berendsen RL, Pieterse CMJ, Bakker PAHM. The rhizosphere microbiome and plant health. Trends Plant Sci. 2012; 17:478–486. 10.1016/j.tplants.2012.04.001 22564542

[24] Pouteau S. Grandbastien MA, Boccara M. Microbial elicitors of plant defence responses activate transcription of a retrotransposon. Plant J. 1994; 5:535–542.

[25] Grandbastien MA, Lucas H, Morel JB, Mhiri C, Vernhettes S, Casacuberta JM. The expression of the tobacco Tnt1 retrotransposon is linked to plant defense responses. Genetica. 1997; 100:241–252. 9440277

[26] Mhiri C, Morel JB, Vernhettes S, Casacuberta JM, Lucas H, Grandbastien MA. The promoter of the tobacco Tnt1 retrotransposon is induced by wounding and by abiotic stress. Plant Mol Biol. 1997; 33:257–266. 9037144 10.1023/a:1005727132202

[27] Mhiri C, De Wit PJGM, Grandbastien MA. Activation of the promoter of the Tnt1 retrotransposon in tomato after inoculation with the fungal pathogen *Cladosporium fulvum*. Mol Plant-Microbe Interactions. 1999; 12:592–603.

[28] Beguiristain T, Grandbastien MA, Puigdomènech P, Casacuberta JM. Three Tnt1 subfamilies show different stress-associated patterns of expression in tobacco. Consequences for retrotransposon control and evolution in plants. Plant Physiol. 2001; 127:212–221. 11553749 10.1104/pp.127.1.212 PMC117977

[29] Melayah D, Bonnivard E. Chalhoub B, Colette A, Grandbastien MA. The mobility of the tobacco Tnt1 retrotransposon correlates with its transcriptional activation by fungal factors. Plant J. 2001; 28:159–168. 11722759 10.1046/j.1365-313x.2001.01141.x

[30] Grandbastien MA, Audeon C, Bonnivard E, Casacuberta JM, Chalhoub B, Costa APP, et al Stress activation and genomic impact of Tnt1 retrotransposons in Solanaceae. Cytogenet Genome Res. 2005; 110:229–241. 10.1159/000084957 16093677

[31] Anca IA, Fromentin J, Bui QT, Mhiri C, Grandbastien MA, Simon-Plas F. Different tobacco retrotransposons are specifically modulated by the elicitor cryptogein and reactive oxygen species. J Plant Physiol. 2014; 171:1533–1540. 10.1016/j.jplph.2014.07.003 25128785

[32] Wicker T, Sabot F, Hua-Van A, Bennetzen JL, Capy P, Chalhoub B, et al A unified classification system for eukaryotic transposable elements. Nature Rev Genet. 2007; 8:973–982. 10.1038/nrg2165 17984973

[33] Kumar A, Bennetzen JL. Plant retrotransposons. Ann Rev Genet. 1999; 33:479–532. 10.1146/annurev.genet.33.1.479 10690416

[34] Natali L, Cossu RM, Mascagni F, Giordani T, Cavallini A. A survey of *Gypsy* and *Copia* LTR-retrotransposon superfamilies and lineages and their distinct dynamics in the *Populus trichocarpa* (L.) genome. Tree Genet Genomes. 2015; 11:107.

[35] Barghini E, Mascagni F, Natali L, Giordani T, Cavallini A. Analysis of the repetitive component and retrotransposon population in the genome of a marine angiosperm, *Posidonia oceanica* (L.) Delile. Mar Genomics. 2015; 24:397–404. 10.1016/j.margen.2015.10.002 26472701

[36] Usai G, Mascagni F, Natali L, Giordani T, Cavallini A. Comparative genome-wide analysis of repetitive DNA in the genus *Populus* L. Tree Genet Genomes. 2017; 13:96.

[37] Buti M, Moretto M, Barghini E, Mascagni F, Natali L, Brilli M, et al The genome sequence and transcriptome of *Potentilla micrantha* and their comparison to *Fragaria vesca* (the woodland strawberry). GigaScience. 2018; 7: 1–14. 10.1093/gigascience/giy010 PMC5893959 29659812

[38] Hawkins JS, Kim HR, Nason JD, Wing RA, Wendel JF. Differential lineage-specific amplification of transposable elements is responsible for genome size variation in *Gossypium*. Genome Res. 2006; 16:1252–1261. 10.1101/gr.5282906 16954538 PMC1581434

[39] Piegu B, Guyot R, Picault N, Roulin A, Saniyal A, Kim H, et al Doubling genome size without polyploidization: Dynamics of retrotransposition-driven genomic expansions in *Oryza australiensis*, a wild relative of rice. Genome Res. 2006; 16:1262–1269. 10.1101/gr.5290206 16963705 PMC1581435

[40] Mascagni F, Giordani T, Ceccarelli M, Cavallini A, Natali L. Genome-wide analysis of LTR-retrotransposon diversity and its impact on the evolution of the genus *Helianthus* (L.). BMC Genomics. 2017; 18:634 10.1186/s12864-017-4050-6 28821238 PMC5563062

[41] Kazazian HH. L1 Retrotransposons shape the mammalian genome. Science. 2000; 289:1152–1153. 10970230 10.1126/science.289.5482.1152

[42] Morgante M, Brunner S, Pea G, Fengler K, Zuccolo A, Rafalski A. Gene duplication and exon shuffling by helitron-like transposons generate intraspecies diversity in maize. Nature Genet. 2005; 37:997 10.1038/ng1615 16056225

[43] Von Sternberg R, Shapiro JA. How repeated retroelements format genome function. Cytogenet Genome Res. 2005; 110:108–116. 10.1159/000084942 16093662

[44] Slotkin RK, Martienssen R. Transposable elements and the epigenetic regulation of the genome. Nature Rev Genet. 2007; 8:272 10.1038/nrg2072 17363976

[45] Badouin H, Gouzy J, Grassa CJ, Murat F, Staton SE, Cottret L, et al The sunflower genome provides insights into oil metabolism, flowering and Asterid evolution. Nature. 2017; 546:148–152. 10.1038/nature22380 28538728

[46] Staton SE, Bakken BH, Blackman BK, Chapman MA, Kane NC, Tang S, et al The sunflower (*Helianthus annuus* L.) genome reflects a recent history of biased accumulation of transposable elements. Plant J. 2012; 72:142–153. 10.1111/j.1365-313X.2012.05072.x 22691070

[47] Natali L, Cossu RM, Barghini E, Giordani T, Buti M, Mascagni F, et al The repetitive component of the sunflower genome as shown by different procedures for assembling next generation sequencing reads. BMC Genomics. 2013; 14:686 10.1186/1471-2164-14-686 24093210 PMC3852528

[48] Mascagni F, Barghini E, Giordani T, Rieseberg LH, Cavallini A, Natali L. Repetitive DNA and plant domestication: variation in copy number and proximity to genes of LTR-Retrotransposons among wild and cultivated sunflower (*Helianthus annuus*) genotypes. Genome Biol Evol. 2015; 7:3368–3382. 10.1093/gbe/evv230 26608057 PMC4700961

[49] Mascagni F, Cavallini A, Giordani T, Natali L. Different histories of two highly variable LTR retrotransposons in sunflower species. Gene. 2017; 634:5–14. 10.1016/j.gene.2017.08.014 28867564

[50] Mascagni F, Vangelisti A, Giordani T, Cavallini A, Natali L. Specific LTR-retrotransposons show copy number variations between wild and cultivated sunflowers. Genes. 2018; 9:433. 10.3390/genes9090433 PMC6162735 30158460

[51] Llorens C, Futami R, Covelli L, Domínguez-Escribá L, Viu JM, Tamarit D, et al The *Gypsy* Database (GyDB) of mobile genetic elements: release 2.0. Nucl Acid Res. 2011; 39:70–74. 10.1093/nar/gkq1061 PMC3013669 21036865

[52] Wright DA, Voytas DF. *Athila*4 of *Arabidopsis* and *Calypso* of Soybean define a lineage of endogenous plant retroviruses. Genome Res. 2002; 12:122–131. 10.1101/gr.196001 11779837 PMC155253

[53] Gorinšek B, Gubensšek F, Kordisš D. Evolutionary genomics of chromoviruses in eukaryotes. Mol Biol Evol. 2004; 21:781–798. 10.1093/molbev/msh057 14739248

[54] Buti M, Giordani T, Vukich M, Gentzbittel L, Pistelli L, Cattonaro F, et al HACRE1, a recently inserted *Copia*-like retrotransposon of sunflower (*Helianthus annuus* L.). Genome. 2009; 52:904–911. 10.1139/g09-064 19935914

[55] Vukich M, Giordani T, Natali L, Cavallini A. *Copia* and *Gypsy* retrotransposons activity in sunflower (*Helianthus annuus* L.). BMC Plant Biol. 2009; 9:150 10.1186/1471-2229-9-150 20030800 PMC2805666

[56] Kawakami T, Dhakal P, Katterhenry AN, Heatherington CA, Ungerer MC. Transposable element proliferation and genome expansion are rare in contemporary sunflower hybrid populations despite widespread transcriptional activity of LTR retrotransposons. Genome Biol Evol. 2011; 3:156–157. 10.1093/gbe/evr005 21282712 PMC3048363

[57] Turrini A, Sbrana C, Avio L, Njeru EM, Bocci G, Bàrberi P, Giovannetti M. Changes in the composition of native root arbuscular mycorrhizal fungal communities during a short-term cover crop-maize succession. Biol Fertility Soils. 2016; 52:643–653.

[58] Leff JW, Lynch RC, Kane NC, Fierer N. Plant domestication and the assembly of bacterial and fungal communities associated with strains of the common sunflower, *Helianthus annuus*. New Phytol. 2017; 214:412–423. 10.1111/nph.14323 27879004

[59] Savary R, Masclaux FG, Wyss T, Droh G, Corella JC, Machado AP, et al A population genomics approach shows widespread geographical distribution of cryptic genomic forms of the symbiotic fungus *Rhizophagus irregularis*. ISME J. 2017; 12:17 10.1038/ismej.2017.153 29027999 PMC5739010

[60] Tisserant E, Malbreil M, Kuo A, Kohler A, Symenoidi A, Balestrini R, et al Genome of an arbuscular mycorrhizal fungus provides insight into the oldest plant symbiosis. Proc Natl Acad Sci. 2013; USA 50:20117–20122. 10.1073/pnas.1313452110 PMC3864322 24277808

[61] Ropars J, Corradi N. Homokaryotic vs heterokaryotic mycelium in arbuscular mycorrhizal fungi: different techniques, different results? New Phytol. 2015; 208:638–641. 10.1111/nph.13448 25952991

[62] Chen ECH, Morin E, Beaudet D, Noel J, Yildirir G, Ndikumana S, et al High intraspecific genome diversity in the model arbuscular mycorrhizal symbiont *Rhizophagus irregularis*. New Phytol. 2018; 220:1161–1171. 10.1111/nph.14989 29355972

[63] Giordani T, Cossu RM, Mascagni F, Marroni F, Morgante M, Cavallini A, Natali L. Genome-wide analysis of LTR-retrotransposons expression in leaves of *Populus x canadensis* water-deprived plants.Tree Genet Genomes. 2016; 12:75.

[64] Phillips JM, Hayman DS. Improved procedures for clearing roots and staining parasitic and vesicular-arbuscular mycorrhizal fungi for rapid assessment of infection. Trans Brit Mycol Soc. 1970; 55:158–161.

[65] Smith SE, Gianinazzi-Pearson V. Phosphate uptake and arbuscular activity in mycorrhizal *Allium cepa* L.: effects of photon irradiance and phosphate nutrition. Austr J Plant Physiol. 1990; 17:177–188.

[66] Giovannetti M, Mosse B. An evaluation of techniques for measuring vesicular arbuscular mycorrhizal infection in roots. New Phytol. 1980; 84:489–500.

[67] Logemann J, Schell J, Willmitzer L. Improved method for the isolation of RNA from plant tissues. Anal Biochem. 1987; 163:16–20. 2441623 10.1016/0003-2697(87)90086-8

[68] Bolger AM, Lohse M, Usadel B. Trimmomatic: a flexible trimmer for Illumina sequence data. Bioinformatics. 2014; 30:2114–2120. 10.1093/bioinformatics/btu170 24695404 PMC4103590

[69] Novák P, Neumann P, Pech J, Steinhaisl J, Macas J. RepeatExplorer: a Galaxy-based web server for genome-wide characterization of eukaryotic repetitive elements from next-generation sequence reads. Bioinformatics. 2013; 29:792–793. 10.1093/bioinformatics/btt054 23376349

[70] Mortazavi A, Williams BA, McCue K, Schaeffer L, Wold B. Mapping and quantifying mammalian transcriptomes by RNA-Seq. Nature Meth. 2008; 5:621–628. 10.1038/nmeth.1226 PMC13303166 18516045

[71] Baggerley K, Deng L, Morris J, Aldaz C. Differential expression in SAGE: accounting for normal between-library variation. Bioinformatics. 2003; 19:1477–1483. 12912827 10.1093/bioinformatics/btg173

[72] Benjamini Y, Hochberg Y. Controlling the false discovery rate: a practical and powerful approach to multiple testing. J Royal Stat Soc. 1995; 57:289–300.

[73] McClintock B. The significance of responses of the genome to challenge. Science. 1984; 226:792–801. 15739260 10.1126/science.15739260

[74] Huettel B, Kanno T, Daxinger L, Bucher E, Van der Winden J, Matzke AJM, Matzke M. RNA-directed DNA methylation mediated by DRD1 and Pol IVb: A versatile pathway for transcriptional gene silencing in plants. Biochim Biophys Acta—Gene Struct Expression. 2007; 1769:358–374. 10.1016/j.bbaexp.2007.03.001 17449119

[75] Lisch D. Epigenetic regulation of transposable elements in plants. Ann Rev Plant Biol. 2009; 60:43–66. 19007329 10.1146/annurev.arplant.59.032607.092744

[76] Volpe TA, Kidner C, Hall IM, Teng G, Grewal SIS, Martienssen RA. Regulation of heterochromatic silencing and histone H3 lysine-9 methylation by RNAi. Science. 2002; 297:1833–1837. 10.1126/science.1074973 12193640

[77] Giordani T, Cavallini A, Natali L. The repetitive component of the sunflower genome. Curr Plant Biol. 2014; 1:45–54.

[78] Yamazaki M, Tsugawa H, Miyao A, Yano M, Wu J, Yamamoto S, et al The rice retrotransposon *Tos17* prefers low-copy-number sequences as integration targets. Mol Genet Genomics. 2001; 265:336–344. 11361345 10.1007/s004380000421

