# Supplementary material for: Arbuscular mycorrhizal fungi induce the expression of specific retrotransposons in roots of sunflower (Helianthus annuus L.)
Source: PLoS One. 2019 Feb 19;14(2):e0212371. doi: 10.1371/journal.pone.0212371 (PMC6380549; doi:10.1371/journal.pone.0212371)
Supplement: S1 Table — (DOC) [file pone.0212371.s001.doc]

## Supporting Information

Article title: **Arbuscular mycorrhizal fungi induce the expression of specific retrotransposons in roots of sunflower (*Helianthus annuus* L.)**

Authors: Alberto Vangelisti, Flavia Mascagni, Tommaso Giordani, Cristiana Sbrana, Alessandra Turrini, Andrea Cavallini, Manuela Giovannetti, Lucia Natali

**S1 Table.** Expression values of 4 house-keeping, 4 glutathione S transferase, 4 peroxidase, and 9 pathogenesis-related (PR) protein encoding genes in sunflower roots during arbuscular mycorrhizal colonization. Three replicates x two treatment durations (4 and 16 days) are reported. For house-keeping genes, the expression level is indicated as high, medium, or low according to qRT-PCR experiments (Vangelisti et al. 2018).

| **ID code** | **Annotation** | **Functional class** | **M4_1 reads per million** | **M4_2 reads per million** | **M4_3 reads per million** | **M16_1 reads per million** | **M16_2 reads per million** | **M16_3 reads per million** |
| --- | --- | --- | --- | --- | --- | --- | --- | --- |
| HanXRQChr05g0138451 | E3 ubiquitin-ligase | housekeeping (highly expressed) | 37.50 | 34.33 | 55.06 | 44.34 | 58.41 | 54.67 |
| HanXRQChr09g0260651 | Oxysterol-binding protein | housekeeping (medium expressed) | 28.38 | 20.32 | 26.57 | 40.49 | 45.81 | 36.65 |
| HanXRQChr15g0464101 | Sugar phosphate translocator | housekeeping (medium expressed) | 29.02 | 27.30 | 22.72 | 25.23 | 28.67 | 23.29 |
| HanXRQChr10g0282971 | Ubiquitin ligase activator of NFKB 1 | housekeeping (lowly espressed) | 17.97 | 14.63 | 20.73 | 23.59 | 25.62 | 18.21 |
| HanXRQChr01g0019851 | glutathione S-transferase | glutathione S transferases | 0.97 | 0.95 | 4.31 | 55.19 | 60.94 | 26.85 |
| HanXRQChr01g0019871 | glutathione S-transferase | glutathione S transferases | 0.00 | 0.06 | 0.00 | 2.61 | 0.23 | 0.99 |
| HanXRQChr06g0183411 | glutathione S-transferase | glutathione S transferases | 0.97 | 1.45 | 3.26 | 184.04 | 119.86 | 92.98 |
| HanXRQChr16g0532301 | glutathione S-transferase | glutathione S transferases | 2.95 | 1.51 | 5.70 | 653.05 | 577.35 | 284.22 |
| HanXRQChr04g0120381 | Cytochrome B5, n2,ATCB5-E,CB5-E isoform 1 | peroxidases | 11.43 | 9.16 | 7.92 | 23.37 | 17.14 | 14.14 |
| HanXRQChr08g0224831 | ascorbate-specific transmembrane electron transporter | peroxidases | 0.38 | 0.22 | 0.50 | 56.04 | 41.59 | 25.79 |
| HanXRQChr10g0318701 | thioredoxin-dependent peroxidase | peroxidases | 77.90 | 83.86 | 134.79 | 145.37 | 95.04 | 150.16 |
| HanXRQChr13g0399961 | peroxiredoxin-2E-2, chloroplastic | peroxidases | 7.62 | 8.21 | 7.33 | 30.06 | 21.92 | 19.72 |
| HanXRQChr03g0085511 | PR-protein | PR-proteins | 21.73 | 27.75 | 37.91 | 405.13 | 192.05 | 254.91 |
| HanXRQChr04g0112801 | chitinase family | PR-proteins | 0.00 | 0.06 | 0.10 | 12.54 | 13.35 | 5.15 |
| HanXRQChr04g0112811 | chitinase family | PR-proteins | 0.00 | 0.06 | 0.02 | 11.84 | 13.30 | 4.66 |
| HanXRQChr04g0112821 | hevamine-A-like | PR-proteins | 0.05 | 0.22 | 0.08 | 8.36 | 8.62 | 4.37 |
| HanXRQChr04g0112831 | hevamine-A-like | PR-proteins | 0.00 | 0.00 | 0.04 | 28.17 | 26.75 | 15.95 |
| HanXRQChr04g0112841 | chitinase family | PR-proteins | 0.00 | 0.22 | 0.16 | 31.43 | 50.68 | 15.64 |
| HanXRQChr04g0112871 | hevamine-A-like | PR-proteins | 0.00 | 0.00 | 0.02 | 1.77 | 1.31 | 0.65 |
| HanXRQChr04g0112881 | chitinase family | PR-proteins | 0.00 | 0.00 | 0.12 | 34.24 | 29.46 | 17.53 |
| HanXRQChr07g0202551 | Disease resistance family LRR family | PR-proteins | 0.32 | 0.28 | 0.28 | 5.18 | 5.81 | 3.26 |
