# Supplementary material for: Arbuscular mycorrhizal fungi induce the expression of specific retrotransposons in roots of sunflower (Helianthus annuus L.)
Source: PLoS One. 2019 Feb 19;14(2):e0212371. doi: 10.1371/journal.pone.0212371 (PMC6380549; doi:10.1371/journal.pone.0212371)
Supplement: S1 Fig — (DOC) [file pone.0212371.s002.doc]

## Supporting Information

Article title: **Arbuscular mycorrhizal fungi induce the expression of specific retrotransposons in roots of sunflower (*Helianthus annuus* L.)**

Authors: Alberto Vangelisti, Flavia Mascagni, Tommaso Giordani, Cristiana Sbrana, Alessandra Turrini, Andrea Cavallini, Manuela Giovannetti, Lucia Natali


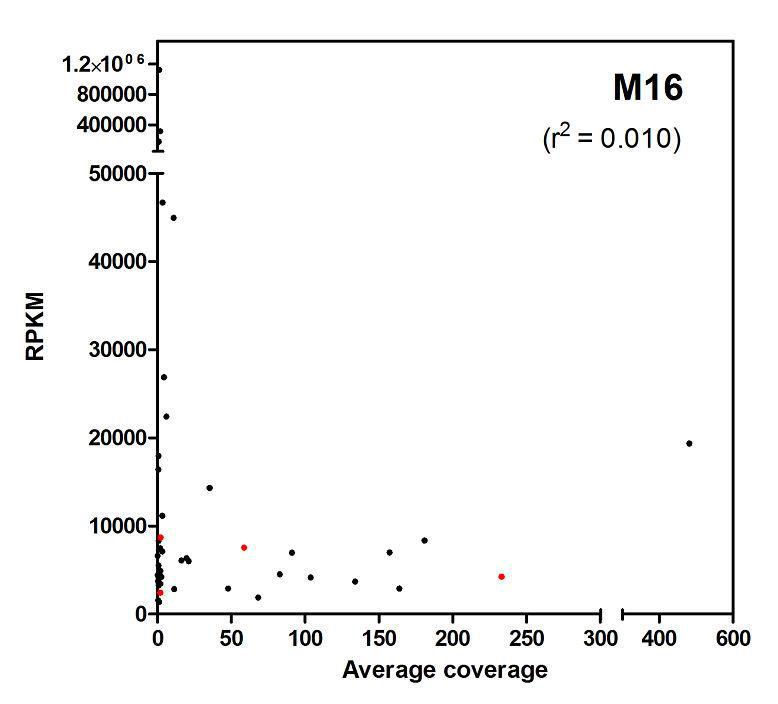


**S1 Fig.** Relationship between RPKM expression values of each of the 46 RT sequences expressed in *H. annuus* M16 roots and the average coverage of the same sequences in the genome. RT sequences differentially expressed between M16 and C16 are indicated in red
